# Association of Advance Care Planning Visits With Intensity of Health Care for Medicare Beneficiaries With Serious Illness at the End of Life

**DOI:** 10.1001/jamahealthforum.2021.1829

**Published:** 2021-07-30

**Authors:** Joel S. Weissman, Amanda J. Reich, Holly G. Prigerson, Priscilla Gazarian, Jennifer Tjia, Dae Kim, Phil Rodgers, Adoma Manful

**Affiliations:** 1 Center for Surgery and Public Health, Brigham and Women’s Hospital, Boston, Massachusetts; 2 Cornell Center for Research on End-of-Life Care, Weill Cornell Medicine, New York, New York; 3 Population and Quantitative Health Sciences, University of Massachusetts Medical School, Worcester; 4 Marcus Institute for Aging Research, Hebrew SeniorLife, Boston, Massachusetts; 5 Department of Family Medicine, University of Michigan, Ann Arbor; 6 Department of Internal Medicine, University of Michigan, Ann Arbor

## Abstract

**Importance:**

Advance care planning (ACP) is intended to maximize the concordance of preferences with end-of-life (EOL) care and is assumed to lead to less intensive use of health care services. The Centers for Medicare & Medicaid Services began reimbursing clinicians for ACP discussions with patients in 2016.

**Objective:**

To determine whether billed ACP visits are associated with intensive use of health care services at EOL.

**Design, Setting, and Participants:**

This prospective patient-level cohort analysis of seriously ill patients included Medicare fee-for-service beneficiaries who met criteria for serious illness from January 1 to December 31, 2016, and died from January 1, 2017, to December 31, 2018. Analyses were completed from November 1, 2020, to March 31, 2021.

**Main Outcomes and Measures:**

Five measures of EOL health care services used (inpatient admission, emergency department visit, and/or intensive care unit stay within 30 days of death; in-hospital death; and timing of first hospice bill) and a measure of EOL expenditures. Analyses were adjusted for age, race and ethnicity, sex, Charlson Comorbidity Index, Medicare-Medicaid dual eligibility, and expenditure by hospital referral region (high, medium, or low). The primary exposure was receipt of a billed ACP service classified as none, timely (>1 month before death), or late (first ACP visit ≤1 month before death).

**Results:**

Of the 955 777 Medicare beneficiaries who met criteria for serious illness in 2016 and died in 2017 or 2018, 522 737 (54.7%) were women, 764 666 (80.0%) were 75 years or older, and 822 684 (86.1%) were non-Hispanic White individuals. Among the study population, 81 131 (8.5%) had a timely ACP visit, and an additional 22 804 (2.4%) had a late ACP visit. After multivariable adjustment, compared with patients without any billed ACP visit, patients with a timely ACP visit experienced significantly less intensive EOL care on 4 of 5 measures, including in-hospital death (adjusted odds ratio [aOR], 0.85; 95% CI, 0.84-0.87), hospital admission (aOR, 0.84; 95% CI, 0.83-0.85), intensive care unit admission (aOR, 0.87; 95% CI, 0.85-0.88), and emergency department visit (OR, 0.83; 95% CI, 0.82-0.84). Only small or insignificant differences in late hospice use or mean total EOL expenditures were noted. Compared with patients without ACP, patients with late ACP experienced more intensive EOL care, including in-hospital death (aOR, 1.22; 95% CI, 1.19-1.26), hospital admission (aOR, 5.28; 95% CI, 5.07-5.50), intensive care unit admission (aOR, 1.57; 95% CI, 1.53-1.62), and emergency department visit (aOR, 3.87; 95% CI, 3.72-4.02).

**Conclusions and Relevance:**

In this cohort study of US Medicare beneficiaries, billed ACP services during the EOL course of patients with serious illness were relatively uncommon, but if they occurred before the last month of life, they were associated with less intensive use of EOL services. Further research on the variables affecting hospice use and expenditures in the EOL period and the differential effect of late ACP is recommended to understand the relative role of ACP in achieving goal-concordant care.

## Key Points

**Question:**

What is the association of a billed advance care planning (ACP) visit with intensive use of health care services at the end of life (EOL) for Medicare beneficiaries with serious illness?

**Findings:**

In this cohort study of claims data of 955 777 Medicare beneficiaries with serious illness who died in 2017 and 2018, billed ACP visits that occurred during the decedents’ EOL course but before the last month of life were relatively uncommon. However, their occurrence was associated with less intensive use of EOL health care services.

**Meaning:**

The findings of this cohort study suggest that ACP is associated with less intensive use of EOL health care services.

## Teaser

This cohort study tests the hypothesis that having a billed advance care planning visit is associated with less intensive use of end-of-life health care services among Medicare beneficiaries with serious illness.

## Introduction

Up to 76% of patients will be unable to participate in decisions about their care at the end of life (EOL).^1,2^ Advance care planning (ACP) is a process that enables the patient and health care system to better align preferences, values, and goals. Advance care planning has been shown to reduce the frequency with which patients die in the hospital^3^ and are admitted to a hospital or an intensive care unit (ICU) in the last 30 days of life.^4,5^ Patients who have engaged in ACP are more likely to enroll in hospice care in a timely fashion,^3,4^ avoiding crisis decisions in the last days before death.

A common ACP product may include documentation of EOL treatment preferences, including advance directives, physician orders for life-sustaining treatment, or assignment of a health care proxy. Advance care planning is intended to aid persons across their life course and may involve multiple points of engagement with clinicians.^6,7^ Advance care planning may be particularly helpful for persons who have a serious illness, who face a heightened risk of death, or whose condition adversely affects their daily quality of life.^8,9^ Although we know that persons living with serious illnesses are more likely than other older patients to have ACP visits,^10^ less is known about how ACP affects their EOL care except among studies of small or local samples^11,12^ and a limited sample of commercially insured patients.^13^

Until recently, the lack of data on ACP visits in Medicare administrative claims limited the ability to examine these phenomena on a population level. In 2016, the Centers for Medicare & Medicaid Services initiated the use of time-based *Current Procedural Terminology* codes (99497 and 99498) to compensate clinicians for having ACP conversations with their patients.^14^ Uptake in the use of these billing codes among Medicare clinicians has been low but is gradually increasing.^15^ Within this context, the goal of this study was to test the hypothesis that having a billed ACP visit for seriously ill patients was associated with less intensive use of health care at EOL.

## Methods

This cohort study was approved by the institutional review board at Partners Healthcare. Data were provided by the Centers for Medicare & Medicaid Services and used under an appropriate data use agreement. The analyses were performed from November 1, 2020, to March 31, 2021, using data from January 1, 2016, to December 31, 2018. The reporting of this study conforms to the Strengthening the Reporting of Observational Studies in Epidemiology (STROBE) reporting guideline for cohort studies.^16^

### Data Source and Study Sample

We used claims from January 1 to December 31, 2016, for all continuously enrolled fee-for-service Medicare beneficiaries, excluding patients with any managed care coverage. Seriously ill patients who were expected to have a median survival of less than 2 years or significant limitations as a result of their disease were prospectively identified using an algorithm that has been used in previous studies and clinical trials.^10,17^ We included patients who had claims that contained the *International Statistical Classification of Diseases and Related Health Problems, Tenth Revision* diagnosis codes of chronic obstructive pulmonary disease and other lung diseases, heart failure, kidney failure, cancer, dementia, and neurodegenerative diseases who also had at least 2 inpatient admissions in the prior 12 months or met the criteria for low body mass index or frailty.^18^ The distribution of these diagnoses in the cohort is available from the authors. Next, we identified beneficiaries 65 years or older who died in 2017 or 2018. Claims for these individual patients were linked with the Medicare beneficiary summary file and inpatient, outpatient, skilled nursing facility, hospice, and carrier files using the beneficiary identification code.

### Measures

#### Use of EOL Health Services

From the Medicare records, we obtained 6 evidence-based measures of use of EOL health care services or intensity^3,19,20,21,22^: (1) in-hospital death, for which hospital discharge status was death; (2) hospital admission, defined as at least 1 inpatient hospital admission within the last 30 days of life; (3) ICU admission, defined as at least 1 admission to the ICU within the last 30 days of life; (4) emergency department (ED) visit, defined as at least 1 ED visit within the last 30 days of life; (5) hospice timing, categorized as no hospice, hospice care initiated at least 4 days before death, or hospice care initiated fewer than 4 days before death; and (6) expenditures, calculated as total Medicare costs for all inpatient, outpatient, skilled nursing facility, and hospice care in the last 30 days of life.

#### ACP Billed Visit

Our primary exposure variable was receipt of a billed ACP visit. Beneficiaries having 1 or more billed visits from January 1, 2016, until death using either the primary code 99497 (first 30 minutes) or the secondary code 99498 (extended time beyond 30 minutes) or both were counted once for the analysis. Beneficiaries with their first ACP visit within 30 days of death were classified separately given that the period of exposure overlapped with the measurement period for EOL outcomes. Thus, we classified decedents as having no ACP (if neither the primary nor the secondary billing code was used in any visit from 2016-2018), timely ACP (if ≥1 ACP claim appeared in any visit from 2016-2018 until 30 days before the death), or late ACP (if the first ACP claim appeared within 30 days before death).

#### Covariates

Covariates were drawn from potential confounders used in previous EOL outcome studies^23,24,25^ and included sex (male or female), age (65-69, 70-74, 75-79, 80-84, 85-89, 90-94, and ≥95 years), Charlson Comorbidity Index (CCI) using a 6-month look-back period from date of death (0, 1, 2, 3, 4, or ≥5), and dual eligibility status for Medicare and Medicaid as a proxy for socioeconomic status. We included race and ethnicity as a covariate because of its association with use of EOL health care services^26,27^ and used the categories as listed in the Master Beneficiary Summary File. Hospital referral regions (HRRs), a standard geographic unit developed by the Dartmouth Institute for Health Policy and Clinical Practice, Hanover, New Hampshire, were used to control for underlying geographic variation in health service use. Total Medicare spending by HRR was calculated as median age-, sex-, and race and ethnicity–adjusted expenses for all Medicare beneficiaries during a 5-year period from 2012 to 2016.^28^ The HRRs were then categorized into high (>75th percentile), medium (25th-75th percentile), and low (<25th percentile) levels of spending. The HRR spending level variable was assigned to each individual beneficiary.

### Statistical Analysis

In unadjusted analyses, we used χ^2^ tests of proportions or 1-way analysis of variance. For analyses adjusting for all covariates, we calculated separate multivariable logistic regression models for binary outcomes to produce the odds ratios (ORs) and 95% CIs of an outcome for timely vs no ACP and late vs no ACP. For EOL expenditures, a multivariable linear regression model was computed to produce adjusted mean differences in expenditure. All *P* values were 2-sided and adjusted using the Bonferroni correction^29^ for multiple hypothesis testing, and *P* < .008 indicated statistical significance.

Because participants who chose to have an ACP visit could be predisposed toward less intensive EOL care regardless of their ACP experience, we performed a matched propensity score adjustment as a sensitivity analysis.^30^ We used a multivariable logistic model to calculate the probability of receiving an ACP (the propensity score) including the aforementioned covariates. The no-ACP group was then matched 1:1 without replacement to the timely ACP group using a greedy matching algorithm.^31^ The estimated propensity score was used for the matching with a caliper size of 0.01. To evaluate the propensity score model, we compared the overlap of propensity scores in each group using graphs (eFigure in the Supplement) and compared propensity score–matched baseline characteristics using standardized differences, with a standardized difference of less than 20% indicating adequate balance between the 2 groups.

## Results

### Sample Characteristics

A total of 955 777 fee-for-service Medicare beneficiaries identified as seriously ill in 2016 died between January 1, 2017, and December 31, 2018 (Table 1). They were more likely to be women (522 737 [54.7%]) and non-Hispanic White individuals (822 684 [86.1%]). Most patients were 75 years or older (764 666 [80.0%]); more than 25% were 90 years or older (256 981 [26.9%]); nearly two-thirds had a CCI of 5 or higher (611 612 [64.0%]); and approximately one-third were dually eligible for Medicare and Medicaid (312 874 [32.7%]).

**Table 1.** Characteristics of Fee-for-Service Medicare Beneficiaries With Serious Illness Who Died in 2017 or 2018, by ACP Grouping

Characteristic	ACP group, No. (%)^a^			
	Total (N = 955 777)	No ACP (n = 851 842)	ACP >30 d before death (n = 81 131)	ACP ≤30 d before death (n = 22 804)
Sex				
Female	522 737 (54.7)	465 286 (54.6)	45 365 (55.9)	12 086 (53.0)
Male	433 040 (45.3)	386 556 (45.4)	35 766 (44.1)	10 718 (47.0)
Age, y				
65-69	76 213 (8.0)	68 553 (8.0)	5869 (7.2)	1791 (7.9)
70-74	114 898 (12.0)	102 615 (12.0)	9461 (11.7)	2822 (12.4)
75-79	141 054 (14.8)	125 621 (14.7)	11 814 (14.6)	3619 (15.9)
80-84	170 171 (17.8)	151 330 (17.8)	14 621 (18.0)	4220 (18.5)
85-89	196 460 (20.6)	174 475 (20.5)	17 139 (21.1)	4846 (21.3)
90-94	168 112 (17.6)	149 566 (17.6)	14 769 (18.2)	3777 (16.6)
≥95	88 869 (9.3)	79 682 (9.4)	7458 (9.2)	1729 (7.6)
Race and ethnicity				
Non-Hispanic White	822 684 (86.1)	734 714 (86.3)	68 535 (84.5)	19 435 (85.2)
Non-Hispanic Black	87 178 (9.1)	77 123 (9.1)	7931 (9.8)	2124 (9.3)
Hispanic	14 634 (1.5)	12 787 (1.5)	1465 (1.8)	382 (1.7)
Asian	12 781 (1.3)	10 934 (1.3)	1471 (1.8)	376 (1.6)
Other^b^	15 194 (1.6)	13 381 (1.6)	1416 (1.7)	397 (1.7)
Unknown	3306 (0.3)	2903 (0.3)	313 (0.4)	90 (0.4)
CCI in the last 6 mo of life				
0	12 396 (1.3)	11 603 (1.4)	761 (0.9)	32 (0.1)
1	49 806 (5.2)	45 906 (5.4)	3526 (4.3)	374 (1.6)
2	79 017 (8.3)	72 471 (8.5)	5647 (7.0)	899 (3.9)
3	94 476 (9.9)	86 014 (10.1)	6983 (8.6)	1479 (6.5)
4	102 809 (10.8)	92 794 (10.9)	8061 (9.9)	1954 (8.6)
≥5	611 612 (64.0)	537 700 (63.1)	55 846 (68.8)	18 066 (79.2)
No claims	5661 (0.6)	5354 (0.6)	307 (0.4)	0
Dual-eligibility status				
No	642 903 (67.3)	572 213 (67.2)	54 719 (67.4)	15 971 (70.0)
Yes	312 874 (32.7)	279 629 (32.8)	26 412 (32.6)	6833 (30.0)
HRR median Medicare spending level				
Low	141 971 (14.9)	127 100 (14.9)	11 337 (14.0)	3534 (15.5)
Medium	527 555 (55.2)	471 855 (55.4)	43 293 (53.4)	12 407 (54.4)
High	286 251 (29.9)	252 887 (29.7)	26 501 (32.7)	6863 (30.1)

Abbreviations: ACP, advance care planning; CCI, Charlson Comorbidity Index; HRR, hospital referral region.

^a^ Percentages have been rounded and may not total 100.

^b^ Specific racial and ethnic categories were unavailable.

Among the cohort, 103 935 patients (10.9%) had at least 1 billed ACP visit; 81 131 (8.5%) had timely ACP visits (first ACP before the last 30 days of life) and 22 804 (2.4%) had late ACP visits (first ACP within the last 30 days of life). Compared with the no-ACP group, Hispanic patients (12 787 [1.5%] vs 1847 [1.8%]), Asian patients (10 934 [1.3%] vs 1847 [1.8%]), patients with more comorbidities (CCI ≥5, 537 700 [63.1%] vs 73 912 [71.1%]), and those living in high-spending HRRs (252 887 [29.7%] vs 33 364 [32.1%]) were somewhat more likely to have had a billed ACP visit (*P* < .001 for all), although the differences were not substantial.

### Association of ACP With EOL Outcomes

#### Unadjusted Results

Compared with patients with no billed ACP visits, patients who had a timely ACP consistently experienced less intensive EOL care, including lower rates of in-hospital death (17 644 [21.8%] vs 201 990 [23.7%]), hospital admission (40 984 [50.5%] vs 448 709 [52.7%]), ICU admission (15 316 [18.9%] vs 173 676 [20.4%]), ED visits (44 171 [54.4%] vs 487 356 [57.2%]), and late hospice referrals (9321 [11.5%] vs 103 699 [12.2%]) (*P* < .001 in pairwise analyses for all) (Figure and eTable 8 in the Supplement). A late ACP was associated with much higher receipt of intensive EOL care (in-hospital death, 6860 [30.1%]; hospital admission, 19 832 [87.0%]; ICU admission, 7095 [31.1%]; ED visit, 19 537 [85.7%]; and late hospice referral, 5275 [23.1%]) (Figure and eTable 8 in the Supplement). Patients with timely ACP visits accounted for slightly higher EOL expenditures ($18 205) than those without an ACP ($17 141), whereas patients with a late ACP had much higher EOL expenditures ($27 187; *P* < .001). This pattern persisted across HRR spending categories (eTable 8 in the Supplement).

**Figure.**
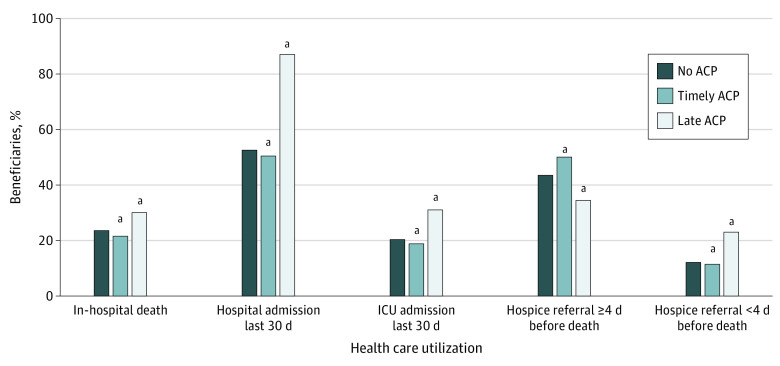
Use of Health Care Services at the End of Life by Fee-for-Service Medicare Beneficiaries With Serious Illness Who Died in 2017 or 2018 Timely advance care planning (ACP) indicates first visit more than 30 days before death; late ACP, first visit within 30 days of death. ICU indicates intensive care unit. ^a^*P* < .001 compared with the no-ACP group.

#### Adjusted Results

In adjusted multivariable analyses (Table 2), seriously ill beneficiaries who had a timely ACP were significantly less likely to die in the hospital (adjusted OR [aOR], 0.85; 95% CI, 0.84-0.87), be admitted to a hospital in their last 30 days of life (aOR, 0.84; 95% CI, 0.83-0.85), have an ICU admission in their last 30 days of life (aOR, 0.87; 95% CI, 0.85-0.88), or have an ED visit in their last 30 days of life (aOR, 0.83; 95% CI, 0.82-0.84). However, decedents with timely ACP visits were somewhat more likely to be enrolled late to hospice (aOR, 1.06; 95% CI, 1.03-1.08). For every measure, decedents with a late ACP experienced more intensive use of EOL services. eTables 1 to 7 in the Supplement show the complete results of multivariable models with all covariates represented.

**Table 2.** Adjusted Odds Ratios for Use of Health Care Resources in Last 30 Days of Life

Measure (n = 950 665)	**aOR** (**95% CI)**	***P* value** ^a^
In-hospital death		
No ACP	1 [Reference]	NA
ACP >30 d before death	0.85 (0.84-0.87)	<.001
ACP ≤30 d before death	1.22 (1.19-1.26)	<.001
Hospital admission in last 30 d		
No ACP	1 [Reference]	NA
ACP >30 d before death	0.84 (0.83-0.85)	<.001
ACP ≤30 d before death	5.28 (5.07-5.50)	<.001
ICU admission in last 30 d		
No ACP	1 [Reference]	NA
ACP >30 d before death	0.87 (0.85-0.88)	<.001
ACP ≤30 d before death	1.57 (1.53-1.62)	<.001
ED visit in last 30 d		
No ACP	1 [Reference]	NA
ACP >30 d before death	0.83 (0.82-0.84)	<.001
ACP ≤30 d before death	3.87 (3.72-4.02)	<.001
Late hospice referral		
No ACP	1 [Reference]	NA
ACP >30 d before death	1.06 (1.03-1.08)	<.001
ACP ≤30 d before death	1.84 (1.78-1.90)	<.001

Abbreviations: ACP, advance care planning; aOR, adjusted odds ratio; ED, emergency department; ICU, intensive care unit; NA, not applicable.

^a^ Bonferroni corrected *P* = .008 for 6 pairwise comparisons. All models were adjusted for sex, age group, race and ethnicity, Charlson Comorbidity Index score, hospital referral region median Medicare spending level, and dual eligibility status.

Differences in adjusted total mean expenditures followed a similar pattern as that of the unadjusted results (Table 3). Compared with patients with no ACP, there were small additional expenditures for patients with a timely ACP in the 2 higher spending regions (mean difference for medium HRR, $245 [95% CI, $46-$445]; mean difference for high HRR, $1411 [95% CI, $1117-$1704]) and large additional expenditures for patients with late ACP (mean difference for medium HRR, $7931 [95% CI, $7573-$8289]; mean difference for high HRR, $10 166 [95% CI, $9615-$10 717]).

**Table 3.** Mean Difference in Expenditure Among HRR Groups

HRR group by ACP (n = 929 505)	Mean difference (95% CI), $	*P* value^a^
All		
No ACP	1 [Reference]	NA
ACP >30 d before death	562 (408 to 716)	<.001
ACP ≤30 d before death	8616 (8337 to 8894)	<.001
HRR median Medicare spending level low		
No ACP	1 [Reference]	NA
ACP >30 d before death	–281 (–695 to 132)	.18
ACP ≤30 d before death	7942 (7231 to 8652)	<.001
HRR median Medicare spending level medium		
No ACP	1 [Reference]	NA
ACP >30 d before death	245 (46 to 445)	.02
ACP ≤30 d before death	7931 (7573 to 8289)	<.001
HRR median Medicare spending level high		
No ACP	1 [Reference]	NA
ACP >30 d before death	1411 (1117 to 1704)	<.001
ACP ≤30 d before death	10 166 (9615 to 10 717)	<.001

Abbreviations: ACP, advance care planning; HRR, hospital referral region; NA, not applicable.

^a^ Bonferroni corrected *P* = .008 for 6 pairwise comparisons. All models adjusted for sex, age group, race and ethnicity, Charlson Comorbidity Index, HRR median Medicare spending level, and dual eligibility status.

#### Sensitivity Analyses Using Propensity Score Methods

After performing the propensity score–matched analysis, the results were nearly identical to those from the traditionally adjusted analyses with 2 exceptions (eTable 9 in the Supplement). There were no longer significant associations between timely ACP and late hospice referral or in mean total expenditures in the medium HRRs.

## Discussion

In this cohort study of US Medicare beneficiaries, we examined the use of EOL health care services among Medicare decedents identified prospectively as seriously ill and found that having timely ACP visits (>30 days before death) with their clinicians was associated with less aggressive care in the last 30 days of life, including fewer hospital deaths, fewer hospital and ICU admissions, and fewer visits to the ED. Timely ACP was associated, albeit slightly, with higher overall EOL expenditures for those living in higher-spending HRRs. Associations between ACP and hospice use varied depending on the comparison. Timely ACP led to timely admission to hospice (≥4 days before death). The association between timely ACP and late hospice use (a typical measure of EOL intensity) was no longer significant after propensity score–matched analysis. The lack of association with late hospice use might be explained by the parallels between hospice use and ACP in the sense that they both may represent actions recognizing the likelihood of death and, at least for some patients, a desire to prioritize quality over quantity of life.

These findings have at least 2 sets of policy implications. First, they address an evolving controversy over the value of ACP. Critics of ACP assert that the limited studies on the benefits of ACP fail to present evidence of changes in the use of health care services or other outcomes.^32^ Our study counters this skepticism with the finding that patients with documented clinician visits involving ACP appear to use care differently at the end of life compared with those without such a visit. Second, patients living with serious illness have a high burden of both physical and psychological symptoms and treatments that adversely affect their quality of life and that of their family members. Advocates of ACP recommend that conversations about values and preferences take place throughout adulthood.^1,33^ However, given limited resources, our results suggest that health systems may prioritize conversations for patients who are closer to death, including those identified as seriously ill. Therefore, understanding the EOL outcomes of these patients is essential. Studies of small, carefully selected cohorts have found that better communication can reduce the use of invasive treatments and can improve bereavement outcomes for care partners.^4,34,35^ Whereas some of these studies have relied on interviews in unique settings, our analysis of Medicare claims, representing visits specifically involving ACP discussions, provides a greater level of standardization to the exposure^36^ and a much larger scale represented by our cohort of all Medicare patients with serious illness.

The disproportionate level of spending at the end of life has often been raised as a reason to more carefully consider how the US provides care.^37,38^ We found a small but significant increase in overall EOL spending for patients with timely ACP visits. Our results conflict with previous studies that have found little or no association between advance directives and health care expenditures in the EOL period.^11,39,40,41,42,43,44^ However, our effect size was small and our sample was large, so this outcome does not necessarily represent a different conclusion. Regardless of the association with health care costs, the effect on patients’ well-being at the end of life due to the intensity of potentially unwanted services is an important measure.^45^

Our findings that patients receiving their first billed ACP visit within 30 days of dying had substantially higher use of health care should serve as a caution for research in this area. There are at least 3 possible explanations for such disparate results when compared with decedents having earlier ACP visits. First, the measurement periods of exposure and outcome overlap. Patients receiving multiple services in the last 30 days of life have more contact with the health care system and more opportunity to have an ACP visit, and this increased contact may have motivated them or their physicians to have a billed ACP conversation. Also, the late ACP visit might have involved a conversation about goals of care or occurred during a hospital stay, which is often associated with high costs. Second, the circumstances of patients who have their first ACP conversation so near to death may be different from those of other decedents (eg, perhaps the reason for the delay is an underlying preference for more life-extending services). Third, patients with late ACP visits may be more likely to be in crisis and thus may decide to have more services performed. Clearly, more studies are needed to determine the dynamics of this subset of Medicare beneficiaries.

### Limitations

Our study has several limitations. First, health care claims do not contain information on the mechanics, content, or quality of the discussion. These discussions could contain preferences for more aggressive rather than less aggressive care. Knowledge of the content of ACP may help explain the contrary findings of late ACP. Second, the uptake of Medicare’s ACP codes has been slow.^15^ In fact, many more patients likely had ACP discussions, but their clinicians did not bill Medicare for the service. Barriers to the adoption and use of these codes may include such factors as clinician lack of awareness of the new codes or unease with documentation requirements, reticence to incur patient cost-sharing, or limits placed on the type of clinical staff eligible to bill.^36^ Third, we lacked data on beneficiaries enrolled in non–fee-for-service Medicare Advantage plans. Because those plans may focus on value, the EOL experiences of that covered population may differ from our findings. Fourth, our findings may be subject to so-called decedent bias.^46^ However, those study designs typically analyze data beginning with the time of death. We identified our cohort of seriously ill patients in 2016 and followed them up until death. Finally, as with all administrative data sets, the findings are subject to the accuracy of the claims.

## Conclusions

Advance care planning has been encouraged for several decades yet has failed to be adopted broadly, and its effect on goal-concordant care at EOL continues to be debated. Medicare’s ACP codes were intended to incentivize ACP by offering clinician compensation. To the extent that many or most patients near EOL desire less aggressive care, the results of this cohort study suggest that the provision of ACP is associated with lower use of health care services. As provision of ACP and the payment of claims continues to grow, future studies using available EOL research methods may be necessary to be confident about its benefits.

## Notes

Supplement. **eTable 1.** Association Between ACP and Hospital Death **eTable 2.** Association Between ACP and Inpatient Admission in the Last 30 Days of Life **eTable 3.** Association Between ACP and ICU Admission in the Last 30 Days of Life **eTable 4.** Association Between ACP and Emergency Department Use in the Last 30 Days of Life **eTable 5.** Association Between ACP and Late Hospice Enrollment **eTable 6.** Association Between ACP and Early Hospice Enrollment **eTable 7.** Association Between ACP and Expenditure in the Last 30 Days of Life by HRR Median Medicare Spending Level **eTable 8.** Health Care Utilization at the End of Life by FFS Medicare Beneficiaries in SI Cohort Who Died in 2017 or 2018 **eTable 9.** Adjusted Odds Ratios for Healthcare Utilization in Last 30 Days of Life Using 1:1 Propensity Score Matched–Beneficiaries **eFigure.** Overlap of Propensity Scores in Seriously Ill Patients Before Matching
