# Supplementary material for: Association of Advance Care Planning Visits With Intensity of Health Care for Medicare Beneficiaries With Serious Illness at the End of Life
Source: JAMA Health Forum. 2021 Jul 30;2(7):e211829. doi: 10.1001/jamahealthforum.2021.1829 (PMC8796875; doi:10.1001/jamahealthforum.2021.1829)
Supplement: Supplement. — eTable 1. Association Between ACP and Hospital Death eTable 2. Association Between ACP and Inpatient Admission in the Last 30 Days of Life eTable 3. Association Between ACP and ICU Admission in the Last 30 Days of Life eTable 4. Association Between ACP and Emergency Department Use in the Last 30 Days of Life eTable 5. Association Between ACP and Late Hospice Enrollment eTable 6. Association Between ACP and Early Hospice Enrollment eTable 7. Association Between ACP and Expenditure in the Last 30 Days of Life by HRR Median Medicare Spending Level eTable 8. Health Care Utilization at the End of Life by FFS Medicare Beneficiaries in SI Cohort Who Died in 2017 or 2018 eTable 9. Adjusted Odds Ratios for Healthcare Utilization in Last 30 Days of Life Using 1:1 Propensity Score Matched–Beneficiaries eFigure. Overlap of Propensity Scores in Seriously Ill Patients Before Matching [file jamahealthforum-e211829-s001.pdf]

Supplementary Online Content

Weissman JS, Reich AJ, Prigerson HG, et al. Association of advance care planning visits with intensity of health care for Medicare beneficiaries with serious illness at the end of life. *JAMA Health Forum*. 2021;2(7):e211829. doi:10.1001/jamahealthforum.2021.1829

eTable 1. Association Between ACP and Hospital Death
eTable 2. Association Between ACP and Inpatient Admission in the Last 30 Days of Life
eTable 3. Association Between ACP and ICU Admission in the Last 30 Days of Life
eTable 4. Association Between ACP and Emergency Department Use in the Last 30 Days of Life
eTable 5. Association Between ACP and Late Hospice Enrollment
eTable 6. Association Between ACP and Early Hospice Enrollment
eTable 7. Association Between ACP and Expenditure in the Last 30 Days of Life by HRR Median Medicare Spending Level
eTable 8. Health Care Utilization at the End of Life by FFS Medicare Beneficiaries in SI Cohort Who Died in 2017 or 2018
eTable 9. Adjusted Odds Ratios for Healthcare Utilization in Last 30 Days of Life Using 1:1 Propensity Score Matched–Beneficiaries
eFigure. Overlap of Propensity Scores in Seriously Ill Patients Before Matching

This supplementary material has been provided by the authors to give readers additional information about their work.

© 2021 Weissman JS et al. *JAMA Health Forum*.

eTable 1. Association Between ACP and Hospital Death				
	**OR**	**95% CI**		***P* value**
**ACP**				
No ACP	ref			
ACP >30d before death	0.854	0.839	0.87	<0.0001
ACP in last 30d	1.221	1.186	1.258	<0.0001
**Age**				
65-69	ref			
70-74	0.904	0.886	0.922	<0.0001
75-79	0.828	0.812	0.844	<0.0001
80-84	0.685	0.672	0.698	<0.0001
85-89	0.559	0.548	0.57	<0.0001
90-94	0.448	0.439	0.457	<0.0001
95+	0.364	0.355	0.373	<0.0001
**Race**				
Non-Hispanic White	ref			
Asian	2.071	1.994	2.152	<0.0001
Hispanic	1.598	1.54	1.658	<0.0001
Non-Hispanic Black	1.346	1.325	1.368	<0.0001
Other	1.534	1.481	1.589	<0.0001
Unknown	1.181	1.094	1.274	<0.0001
**Sex**				
Male	ref			
Female	0.99	0.981	1	0.0562
**Charlson Comorbidity Score**				
0	ref			
1	1.529	1.408	1.661	<0.0001
2	2.205	2.037	2.387	<0.0001
3	3.06	2.83	3.309	<0.0001
4	3.815	3.529	4.124	<0.0001
5+	5.179	4.798	5.59	<0.0001
**HRR Median Medicare Spending Level**				
Medium	ref			
Low	1.169	1.156	1.182	<0.0001
High	1.042	1.027	1.057	<0.0001
**Dual Eligibility Status**				
No	ref			
Yes	0.85	0.841	0.859	<0.0001

© 2021 Weissman JS et al. *JAMA Health Forum*.

eTable 2. Association Between ACP and Inpatient Admission in the Last 30 Days of Life				
	**OR**	**95% CI**		***P* value**
**ACP**				
No ACP	ref			
ACP >30d before death	0.84	0.827	0.853	<0.0001
ACP in last 30d	5.275	5.069	5.49	<0.0001
**Age**				
65-69	ref			
70-74	0.956	0.937	0.975	<0.0001
75-79	0.9	0.883	0.918	<0.0001
80-84	0.79	0.775	0.805	<0.0001
85-89	0.682	0.67	0.695	<0.0001
90-94	0.582	0.571	0.593	<0.0001
95+	0.484	0.474	0.494	<0.0001
**Race**				
Non-Hispanic White	ref			
Asian	1.619	1.558	1.683	<0.0001
Hispanic	1.49	1.438	1.545	<0.0001
Non-Hispanic Black	1.275	1.256	1.295	<0.0001
Other	1.339	1.293	1.387	<0.0001
Unknown	1.078	1	1.161	0.0488
**Sex**				
Male	ref			
Female	1.011	1.002	1.02	0.0123
**Charlson Comorbidity Score**				
0	ref			
1	2.114	1.981	2.256	<0.0001
2	3.41	3.203	3.631	<0.0001
3	5.158	4.847	5.489	<0.0001
4	7.14	6.711	7.597	<0.0001
5+	13.36	12.571	14.198	<0.0001
**HRR Median Medicare Spending Level**				
Medium	ref			
Low	1.209	1.198	1.221	<0.0001
High	0.901	0.89	0.913	<0.0001
**Dual Eligibility Status**				
No	ref			
Yes	0.773	0.766	0.781	<0.0001

© 2021 Weissman JS et al. *JAMA Health Forum*.

eTable 3. Association Between ACP and ICU Admission in the Last 30 Days of Life				
	**OR**	**95% CI**		***P* value**
**ACP**				
No ACP	ref			
ACP >30d before death	0.866	0.849	0.882	<0.0001
ACP in last 30d	1.57	1.525	1.617	<0.0001
**Age**				
65-69	ref			
70-74	0.891	0.873	0.909	<0.0001
75-79	0.776	0.76	0.791	<0.0001
80-84	0.604	0.592	0.616	<0.0001
85-89	0.455	0.446	0.464	<0.0001
90-94	0.309	0.302	0.315	<0.0001
95+	0.207	0.201	0.214	<0.0001
**Race**				
Non-Hispanic White	ref			
Asian	1.809	1.736	1.885	<0.0001
Hispanic	1.617	1.555	1.681	<0.0001
Non-Hispanic Black	1.319	1.297	1.341	<0.0001
Other	1.328	1.278	1.379	<0.0001
Unknown	1.078	0.995	1.167	0.0647
**Sex**				
Male	ref			
Female	0.974	0.964	0.985	<0.0001
**Charlson Comorbidity Score**				
0	ref			
1	1.689	1.524	1.871	<0.0001
2	2.622	2.376	2.893	<0.0001
3	3.716	3.372	4.095	<0.0001
4	4.838	4.393	5.328	<0.0001
5+	6.989	6.354	7.687	<0.0001
**HRR Median Medicare Spending Level**				
Medium	ref			
Low	1.258	1.244	1.273	<0.0001
High	0.842	0.828	0.855	<0.0001
**Dual Eligibility Status**				
No	ref			
Yes	0.88	0.87	0.891	<0.0001

© 2021 Weissman JS et al. *JAMA Health Forum*.

**eTable 4. Association Between ACP and Emergency Department Use in the Last 30 Days of Life**				
	**OR**	**95% CI**		***P* value**
**ACP**				
No ACP	ref			
ACP >30d before death	0.829	0.817	0.842	<0.0001
ACP in last 30d	3.869	3.724	4.018	<0.0001
**Age**				
65-69	ref			
70-74	0.951	0.932	0.97	<0.0001
75-79	0.907	0.89	0.925	<0.0001
80-84	0.825	0.81	0.841	<0.0001
85-89	0.729	0.716	0.743	<0.0001
90-94	0.642	0.63	0.655	<0.0001
95+	0.539	0.528	0.551	<0.0001
**Race**				
Non-Hispanic White	ref			
Asian	1.501	1.445	1.561	<0.0001
Hispanic	1.424	1.373	1.476	<0.0001
Non-Hispanic Black	1.34	1.32	1.362	<0.0001
Other	1.147	1.108	1.188	<0.0001
Unknown	1.04	0.966	1.119	0.3028
**Sex**				
Male	ref			
Female	0.972	0.964	0.981	<0.0001
**Charlson Comorbidity Score**				
0	ref			
1	1.345	1.281	1.413	<0.0001
2	1.919	1.831	2.011	<0.0001
3	2.693	2.571	2.822	<0.0001
4	3.562	3.401	3.731	<0.0001
5+	5.948	5.687	6.221	<0.0001
**HRR Median Medicare Spending Level**				
Medium	ref			
Low	1.148	1.137	1.159	<0.0001
High	0.952	0.94	0.964	<0.0001
**Dual Eligibility Status**				
No	ref			
Yes	0.816	0.808	0.823	<0.0001

© 2021 Weissman JS et al. *JAMA Health Forum*.

eTable 5. Association Between ACP and Late Hospice Enrollment				
	**OR**	**95% CI**		***P* value**
**ACP**				
No ACP	ref			
ACP >30d before death	1.057	1.031	1.083	<0.0001
ACP in last 30d	1.839	1.776	1.904	<0.0001
**Age**				
65-69	ref			
70-74	1.198	1.162	1.236	<0.0001
75-79	1.351	1.311	1.391	<0.0001
80-84	1.565	1.521	1.611	<0.0001
85-89	1.786	1.736	1.838	<0.0001
90-94	2.01	1.952	2.07	<0.0001
95+	2.099	2.029	2.171	<0.0001
**Race**				
Non-Hispanic White	ref			
Asian	0.572	0.537	0.609	<0.0001
Hispanic	0.825	0.781	0.872	<0.0001
Non-Hispanic Black	0.563	0.548	0.577	<0.0001
Other	0.68	0.643	0.718	<0.0001
Unknown	0.934	0.835	1.044	0.2292
**Sex**				
Male	ref			
Female	1.113	1.098	1.128	<0.0001
**Charlson Comorbidity Score**				
0	ref			
1	3.62	3.214	4.077	<0.0001
2	4.672	4.162	5.245	<0.0001
3	5.449	4.859	6.11	<0.0001
4	6.13	5.469	6.87	<0.0001
5+	7.98	7.132	8.93	<0.0001
**HRR Median Medicare Spending Level**				
Medium	ref			
Low	1.053	1.037	1.068	<0.0001
High	0.747	0.732	0.762	<0.0001
**Dual Eligibility Status**				
No	ref			
Yes	0.769	0.757	0.78	<0.0001

© 2021 Weissman JS et al. *JAMA Health Forum*.

eTable 6. Association Between ACP and Early Hospice Enrollment				
	**OR**	**95% CI**		***P* value**
**ACP**				
No ACP	ref			
ACP >30d before death	1.333	1.312	1.354	<0.0001
ACP in last 30d	0.852	0.826	0.878	<0.0001
**Age**				
65-69	ref			
70-74	1.204	1.18	1.229	<0.0001
75-79	1.39	1.363	1.418	<0.0001
80-84	1.7	1.668	1.732	<0.0001
85-89	2.017	1.979	2.055	<0.0001
90-94	2.35	2.305	2.396	<0.0001
95+	2.599	2.543	2.657	<0.0001
**Race**				
Non-Hispanic White	ref			
Asian	0.584	0.561	0.607	<0.0001
Hispanic	0.848	0.818	0.879	<0.0001
Non-Hispanic Black	0.728	0.717	0.74	<0.0001
Other	0.675	0.652	0.7	<0.0001
Unknown	0.873	0.809	0.943	0.0005
**Sex**				
Male	ref			
Female	1.261	1.25	1.273	<0.0001
**Charlson Comorbidity Score**				
0	ref			
1	3.07	2.931	3.216	<0.0001
2	3.135	2.997	3.278	<0.0001
3	2.832	2.709	2.96	<0.0001
4	2.553	2.442	2.668	<0.0001
5+	2.401	2.301	2.505	<0.0001
**HRR Median Medicare Spending Level**				
Medium	ref			
Low	0.943	0.934	0.953	<0.0001
High	0.832	0.822	0.843	<0.0001
**Dual Eligibility Status**				
No	ref			
Yes	0.853	0.844	0.861	<0.0001

© 2021 Weissman JS et al. *JAMA Health Forum*.

eTable 7. Association Between ACP and Expenditure in the Last 30 Days of Life by HRR Median Medicare Spending Level				
**HRR Median Medicare Spending Level = Low**				
	**Mean Difference**	**95% CI**		***P* value**
**ACP**				
No ACP	ref			
ACP >30d before death	7941.89	7231.36	8652.42	<.0001
ACP in last 30d	-281.45	-695.06	132.16	0.1823
**Age**				
65-69	ref			
70-74	-2375.22	-2903.23	-1847.22	<.0001
75-79	-3861.58	-4371	-3352.15	<.0001
80-84	-6888.64	-7381.39	-6395.89	<.0001
85-89	-9248.52	-9731.99	-8765.05	<.0001
90-94	-11011	-11507	-10515	<.0001
95+	-11696	-12251	-11141	<.0001
**Race**				
Asian	ref			
Hispanic	-516.97	-1777.62	743.68	0.4215
Non-Hispanic Black	-1467.93	-2488.95	-446.9	0.0048
Other	-2949.43	-3898.4	-2000.45	<.0001
Unknown	-3644.12	-5559	-1729.25	0.0002
Non-Hispanic White	-7124.45	-7868.12	-6380.78	<.0001
**Sex**				
Female	ref			
Male	275.72	44.8294	506.61	0.0193
**Charlson Comorbidity Score**				
0	ref			
1	3212.37	1984.43	4440.32	<.0001
2	5029.91	3841.02	6218.8	<.0001
3	7039.78	5863.94	8215.63	<.0001
4	8563.57	7392.55	9734.6	<.0001
5+	12905	11772	14039	<.0001
**Dual Eligibility Status**				
No	ref			
Yes	-2445.01	-2695.94	-2194.09	<.0001
**HRR Median Medicare Spending Level= Medium**				
	**Mean Difference**	**95% CI**		***P* value**
**ACP**				
No ACP	ref			

© 2021 Weissman JS et al. *JAMA Health Forum*.

ACP >30d before death	7930.64	7572.59	8288.68	<.0001
ACP in last 30d	245.35	45.8168	444.89	0.016
**Age**				
65-69	ref			
70-74	-2044.13	-2294.28	-1793.98	<.0001
75-79	-3693.34	-3934.84	-3451.84	<.0001
80-84	-6478.35	-6713.44	-6243.26	<.0001
85-89	-8490.47	-8722.04	-8258.9	<.0001
90-94	-10143	-10381	-9904.06	<.0001
95+	-11186	-11458	-10915	<.0001
**Race**				
Asian	ref			
Hispanic	-3823.96	-4653.41	-2994.51	<.0001
Non-Hispanic Black	-5800.66	-6439.07	-5162.24	<.0001
Other	-5634.27	-6437.76	-4830.78	<.0001
Unknown	-8630.32	-9797.73	-7462.92	<.0001
Non-Hispanic White	-9834.6	-10452	-9216.9	<.0001
**Sex**				
Female	ref			
Male	387.33	273.79	500.88	<.0001
**Charlson Comorbidity Score**				
0	ref			
1	3082.02	2432.26	3731.79	<.0001
2	4614.22	3987.25	5241.18	<.0001
3	6471.74	5851.01	7092.47	<.0001
4	8117.91	7499.79	8736.03	<.0001
5+	13086	12487	13685	<.0001
**Dual Eligibility Status**				
No	ref			
Yes	-2422.73	-2545.79	-2299.68	<.0001
**HRR Median Medicare Spending Level = High**				
	**Mean Difference**	**95% CI**		***P* value**
**ACP**				
No ACP	ref			
ACP >30d before death	10166	9615.28	10717	<.0001
ACP in last 30d	1410.64	1116.82	1704.46	<.0001
**Age**				
65-69	ref			
70-74	-1461.6	-1853.76	-1069.44	<.0001
75-79	-3535.6	-3913.74	-3157.46	<.0001

© 2021 Weissman JS et al. *JAMA Health Forum*.

80-84	-5856.55	-6223.68	-5489.42	<.0001
85-89	-7968.46	-8329.66	-7607.26	<.0001
90-94	-9933.27	-10305	-9561.71	<.0001
95+	-10979	-11400	-10557	<.0001
**Race**				
Asian	ref			
Hispanic	-4489.41	-5323.02	-3655.81	<.0001
Non-Hispanic Black	-6321.08	-7020.73	-5621.44	<.0001
Other	-5303.61	-6260.55	-4346.68	<.0001
Unknown	-6402.8	-7944.14	-4861.46	<.0001
Non-Hispanic White	-10187	-10850	-9524.2	<.0001
**Sex**				
Female	ref			
Male	979.45	803.17	1155.72	<.0001
**Charlson Comorbidity Score**				
0	ref			
1	2767.48	1758.82	3776.13	<.0001
2	4767.75	3794.28	5741.22	<.0001
3	6625.4	5662.11	7588.69	<.0001
4	8834.09	7875.23	9792.96	<.0001
5+	15345	14418	16271	<.0001
**Dual Eligibility Status**				
No	ref			
Yes	-1037.69	-1224.9	-850.48	<.0001

© 2021 Weissman JS et al. *JAMA Health Forum*.

eTable 8. Health Care Utilization at the End of Life by FFS Medicare Beneficiaries in SI Cohort Who Died in 2017 or 2018*									
	**Total**	**% or SD**	**No ACP**	**% or SD**	**ACP > 30d before death**	**% or SD**	**ACP in last 30 d**	**% or SD**	***P* value**
**Total N**	955,777	100.00%	851,842	89.10%	81,131	8.50%	22,804	2.40%	
**In Hospital Death**									
No	729,283	76.30%	649,852	76.30%	63,487	78.30%	15,944	69.90%	<0.0001
Yes	226,494	23.70%	201,990	23.70%	17,644	21.70%	6,860	30.10%	
**Total N**	955,777	100.00%	851,842	100.00%	81,131	100.00%	22,804	100.00%	
**Hospital Admission, last 30 days**									
No	446,252	46.70%	403,133	47.30%	40,147	49.50%	2,972	13.00%	<0.0001
Yes	509,525	53.30%	448,709	52.70%	40,984	50.50%	19,832	87.00%	
**Total N**	955,777	100.00%	851,842	100.00%	81,131	100.00%	22,804	100.00%	
**ICU admission, last 30 days**									
No	759,690	79.50%	678,166	79.60%	65,815	81.10%	15,709	68.90%	<0.0001
Yes	196,087	20.50%	173,676	20.40%	15,316	18.90%	7,095	31.10%	
**Total N**	955,777	100.00%	851,842	100.00%	81,131	100.00%	22,804	100.00%	
**ED visit, last 30 days**									
No	404,713	42.30%	364,486	42.80%	36,960	45.60%	3,267	14.30%	<0.0001
Yes	551,064	57.70%	487,356	57.20%	44,171	54.40%	19,537	85.70%	
**Total N**	955,777	100.00%	851,842	100.00%	81,131	100.00%	22,804	100.00%	
**Timing of Hospice Referral**									
No Hospice	418,499	43.80%	377,607	44.30%	31,236	38.50%	9,656	42.30%	<0.0001
Hospice ≥ 4 days before death	418,983	43.80%	370,536	43.50%	40,574	50.00%	7,873	34.50%	
Hospice < 4 days before death	118,295	12.40%	103,699	12.20%	9,321	11.50%	5,275	23.10%	

© 2021 Weissman JS et al. *JAMA Health Forum*.

**Total N**	955,777	100.00%	851,842	100.00%	81,131	100.00%	22,804	100.00%	
**EOL Expenditure in the last 30 days**									
Mean (SD)	$17,481	$22,029	$17,141	$21,876	$18,205	$22,785	$27,187	$24,742	<0.0001
**Total N**	929,502		827,325		79,376		22,804		
**EOL Expenditure in the last 30 d stratified by HRR Median Medicare Spending Level**									
Mean, SD (HRR Level=Low)	$16,332	$22,010	$16,082	$21,837	$16,196	$22,991	$25,441	$24,673	<0.0001
Mean, SD (HRR Level=Medium)	$16,582	$20,878	$16,296	$20,797	$16,986	$21,127	$25,802	$22,922	<0.0001
Mean, SD (HRR Level=High)	$19,703	$23,867	$19,244	$23,644	$21,508	$24,943	$31,059	$27,404	<0.0001
**Total N**	929,502		827,325		79,376		22,804		
ACP: Advance Care Planning									
FFS: Fee For Service									
SI: Seriously Ill									
HRR: Hospital Referral Region									

© 2021 Weissman JS et al. *JAMA Health Forum*.

**eTable 9. Adjusted Odds Ratios for Healthcare Utilization in Last 30 Days of Life Using 1:1 Propensity Score Matched–Beneficiaries***			
	**aOR**	**95% CI**	**pvalue**
**In Hospital Death**			
No ACP	ref		
Yes ACP before last 30d	0.86	80.84-0.88	<0.0001
**Admission in the last 30 d**			
No ACP	ref		
Yes ACP before last 30d	0.82	0.80-0.84	<0.0001
**ICU visit in the last 30 d**			
No ACP	ref		
Yes ACP before last 30d	0.86	0.84-0.88	<0.0001
**ED visit in the last 30 d**			
No ACP	ref		
Yes ACP before last 30d	0.81	0.80-0.83	<0.0001
**Late Hospice Referral**			
No ACP	ref		
Yes ACP before last 30d	0.99	0.96-1.04	0.76
**Total N**	161,672		
*all models adjusted for sex, age group, race/eth, CCI score, HRR cost class and dual eligibility status, using propensity score matched beneficiaries			
**Mean Difference in Expenditure**		**95% CI**	**pvalue**
No ACP	ref		
Yes ACP before last 30d	$565	($356-$773)	<0.0001
**Total N**	161,672		
**Mean Difference in Expenditure**		**95% CI**	**pvalue**
**HRR Median Medicare Spending Level =Low**			
No ACP	ref		
Yes ACP before last 30d	-$268	($-817-$282)	0.33
**HRR Median Medicare Spending Level =Medium**			
No ACP	ref		
Yes ACP before last 30d	$226	($-44-$495)	0.10
**HRR Median Medicare Spending Level=High**			
No ACP	ref		
Yes ACP before last 30d	$1,468	($1073-$1864)	<0.0001

© 2021 Weissman JS et al. *JAMA Health Forum*.

**Total N**	161,672		
*Bonferroni Corrected P =0.008, for multiple 6 pairwise comparisons			
*expenditure stratified by Median Medicare Spending Level by HRR, class models adjusted for sex, age group, race/eth, CCI score, and dual eligibility status, using propensity score matched beneficiaries			
PS match = 99.6% match			

© 2021 Weissman JS et al. *JAMA Health Forum*.

eFigure. Overlap of Propensity Scores in Seriously Ill Patients Before Matching

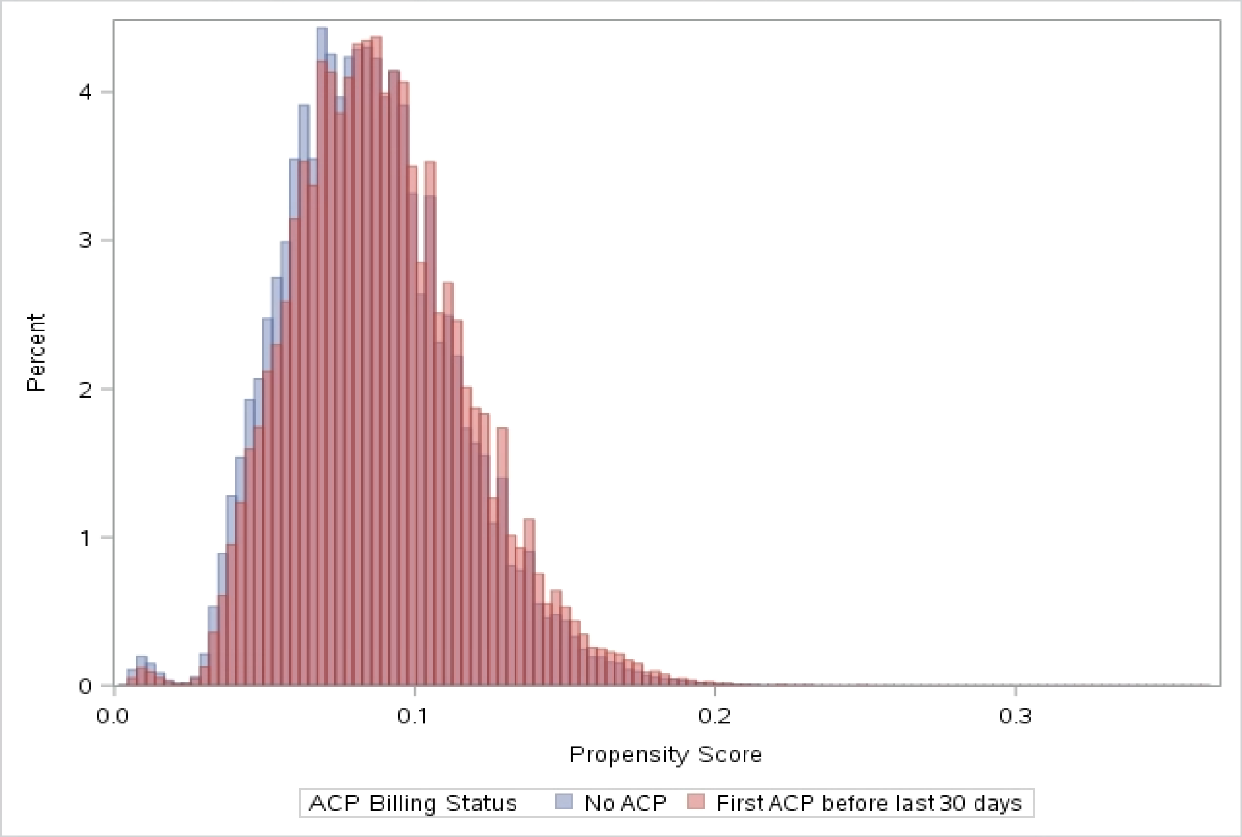

© 2021 Weissman JS et al. *JAMA Health Forum*.
